# Use of Contemporary Groups in the Construction of Multi-Environment Trial Datasets for Selection in Plant Breeding Programs

**DOI:** 10.3389/fpls.2020.623586

**Published:** 2021-02-02

**Authors:** Alison Smith, Aanandini Ganesalingam, Christopher Lisle, Gururaj Kadkol, Kristy Hobson, Brian Cullis

**Affiliations:** ^1^Centre for Bioinformatics and Biometrics, School of Mathematics and Applied Statistics, National Institute for Applied Statistics Research Australia, University of Wollongong, Wollongong, NSW, Australia; ^2^Centre for Bioinformatics and Biometrics, School of Mathematics and Applied Statistics, National Institute for Applied Statistics Research Australia, University of Wollongong, Wollongong, NSW, Australia; ^3^New South Wales Department of Primary Industries, Tamworth Agricultural Institute, Calala, NSW, Australia

**Keywords:** multi-environment trials, linear mixed models, model-based design, contemporary groups, selection, plant breeding

## Abstract

Plant breeding programs use multi-environment trial (MET) data to select superior lines, with the ultimate aim of increasing genetic gain. Selection accuracy can be improved with the use of advanced statistical analysis methods that employ informative models for genotype by environment interaction, include information on genetic relatedness and appropriately accommodate within-trial error variation. The gains will only be achieved, however, if the methods are applied to suitable MET datasets. In this paper we present an approach for constructing MET datasets that optimizes the information available for selection decisions. This is based on two new concepts that characterize the structure of a breeding program. The first is that of “contemporary groups,” which are defined to be groups of lines that enter the initial testing stage of the breeding program in the same year. The second is that of “data bands,” which are sequences of trials that correspond to the progression through stages of testing from year to year. MET datasets are then formed by combining bands of data in such a way as to trace the selection histories of lines within contemporary groups. Given a specified dataset, we use the A-optimality criterion from the model-based design literature to quantify the information for any given selection decision. We demonstrate the methods using two motivating examples from a durum and chickpea breeding program. Datasets constructed using contemporary groups and data bands are shown to be superior to other forms, in particular those that relate to a single year alone.

## 1. Introduction

Plant breeding is a process that consists of methods for the creation, selection, and fixation of superior plants in terms of productivity or quality (Moose and Mumm, 2008). During this process, the ability to select the best lines and discard others is critical in constantly improving the breeding gene pool (Zamir, 2001). Generally, breeding programs have cycle lengths that span 8 to 10 years; that is from initial cross to variety commercialization. The majority of programs follow the modified pedigree breeding method, where traits with high heritability are selected first, and traits of lower heritability selected in later generations when lines become fixed (Collard and Mackill, 2008). The early population stages in such programs are often focused on several traits of commercial importance, including disease resistance, herbicide tolerance, phenology type, and functional grain quality. At the advanced evaluation stages selection is focused on grain yield across target production environments (TPE), to appropriately gauge genotype by environment interaction (G×E), as genotypes vary in their response to different environments. Note that in this paper the terms “genotype” and “line” are used synonymously.

While there are multiple traits of interest under selection, yield is often the trait of foremost interest. In order to achieve efficiency in yield selection, yield data is generated from a series of field trials across years (synonymous with seasons) and geographical locations, known as multi-environment trials (METs). METs are an essential evaluation tool in plant breeding programs, as they enable an effective measure of G×E. This is particularly important in the Australian agricultural environment, which is known to be extremely variable between locations and seasons (Chapman et al., 2003). As a result, advanced evaluation stages in breeding programs use expanded numbers of evaluation environments to appropriately assess across all TPE.

**Figure 1:**
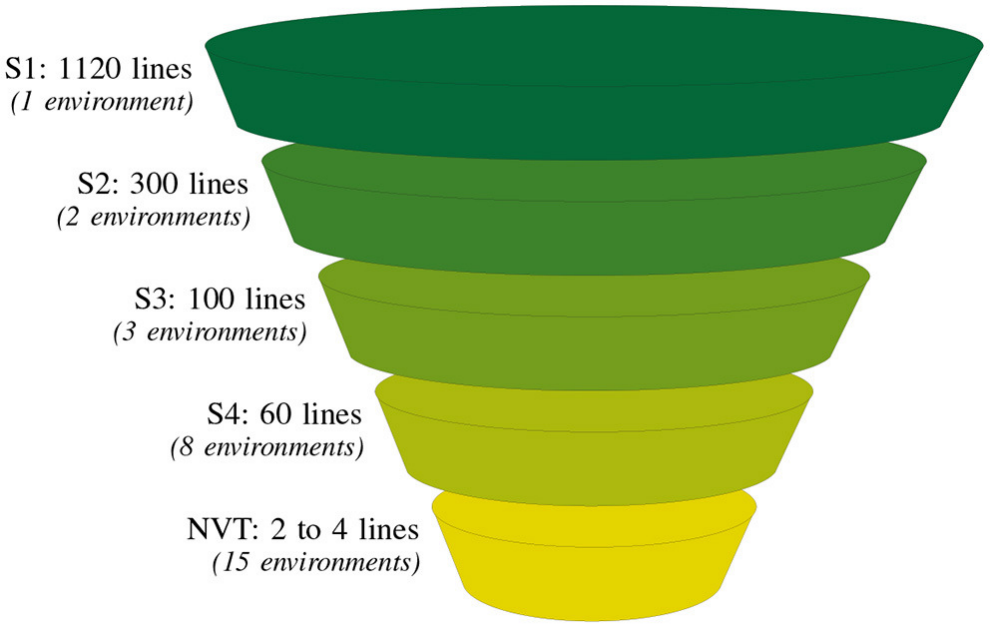
Summary of the typical number of lines and environments (in parentheses) at each testing stage for one of our motivating examples, the durum breeding program. There are four breeding stages of testing: stage 1 (S1) to stage 4 (S4) and a final stage corresponding to the independent Australian testing system, the National Variety Trials (NVT).

The breeding process can be viewed as a multi-year, multi-cycle collection of data (Arief et al., 2019). In particular, we find that programs are underpinned by a grouping of lines that are derived together from a fixed number of crosses at the crossing block stage. These lines are essentially “born” together and are subsequently tested together. This cohort of lines then progress through the bulk population stages, are derived to fixed lines and tested sequentially in the advanced evaluation stages. We refer to these broad grouping of lines as “contemporary groups” (CGs). As an example we consider the Durum Breeding Australia (DBA) program from one of our motivating examples (see section 3) in which there are four stages of testing (denoted S1 to S4). Within the time-frame 2015 to 2018, the following four CGs were created: CG15, CG16, CG17, and CG18 corresponding to lines in S1 trials in those years. Then, for example, a subset of the lines from CG15 was progressed to S2 trials in 2016 and so on to S3 in 2017 and finally to S4 in 2018. As lines progress through stages they decrease in number, similar to the narrowing observed in the funnel structure seen in Figure 1. Following on from this figure, four selection decisions would be made annually on S1, S2, S3, and S4 lines as they progress to the next stage of testing. The final stage selection decision, that is, for S4, is concerned with the submission of elite lines to the Australian national crop variety testing program, the National Variety Trials (NVT).

For plant breeding programs in Australia, the preferred approach for the analysis of MET data is the single stage Factor Analytic Linear Mixed Model (FALMM) of Smith et al. (2001), with modeling of spatial variation for individual trials (Gilmour et al., 1997; Stefanova et al., 2009). This analysis approach is also the preferred method of analysis for the NVT (Smith and Cullis, 2018). Oakey et al. (2007) extended these models with the inclusion of information on genetic relatedness through ancestral information (pedigree), which Beeck et al. (2010) utilized to enable partitioning of additive and non-additive effects for selection in a canola breeding program.

There is a substantial amount of literature demonstrating the improved selection accuracy resulting from the use of a one stage FALMM that incorporates pedigree (ancestry) information (see Oakey et al., 2007; Beeck et al., 2010; Smith and Cullis, 2018; Ukrainetz et al., 2018). The inclusion of pedigree information via the numerator relationship matrix (NRM) provides links between genotypes both within and between environments. This enables more reliable estimation of genetic variance parameters and thence more accurate predictions of total genetic effects as required for selection.

It is well-known that these developments in methods of analysis contribute to genetic gains, as shown by the wide-spread adoption of the FALMM of Smith et al. (2001) in Australian plant breeding programs (Gogel et al., 2018). In Table 1, we have compiled a concise summary of literature specific to plant breeding METs and the methods of analysis. In the process of compiling this summary, we noted that there are very few papers written on the construction of the underlying MET datasets to realize these genetic gains from an analysis point of view. For example, the paper by Arief et al. (2019) indicates that a MET dataset across stages of a breeding program in a combined analysis is the most advantageous. However, they used a two-stage linear mixed model without the inclusion of pedigree information. In contrast, Yan and Rajcan (2003) used a similar approach (single stage, without pedigree) and found that a single year MET dataset was sufficient to identify the best and worst selections. Despite the vast amounts of data breeding programs generate, it is clear that there is little consensus on the construction of datasets for selection decisions in the current literature.

**Table 1:** Summary of studies based on plant breeding METs, their dataset composition, and methods of analysis.

**Crop - trait**	**Selection?**	**Aim**	**Dataset**	**Analysis**	**References**
Barley & Wheat, GY	N	Present an approach for the analysis of early stage breeding METs	Barley S3 comprising 125 lines at 3 locations in 1992 & Wheat early generation yield trials sown at 3 environments in 1991	1 & 2 stage LMM	Cullis et al., 1998
Oat, GY	N	Identify long-term sources of variation	S4 trials of 10-20 lines across 22 locations across 2 years, (174 trials spanning 1985 to 1994)	2 stage LMM	Frensham et al., 1998
Navy beans, GY	N	Report the results of Pattern analysis on TPE	MET comprising 15 locations across the years 1983 to 1989	VCLMM	Redden et al., 2000
Soy bean, GY	N	Evaluate multi-year data vs. single year data of variety performance	MET dataset comprising variety trials at 4 locations per year, from 1991 to 2000	LMM	Yan and Rajcan, 2003
Wheat, GY	N	Review the principles of biplot analysis for MET data	MET dataset comprising 18 winter wheat varieties tested at 9 Ontario locations in 1993	LMM	Yan and Tinker, 2006
Wheat, GY	Y	Identify relevant testing environments and improve predictive value of data	MET comprising: 22 varieties evaluated in 32 environments	LMM	Thomason and Phillips, 2006
Sugarcane	Y	Demonstrate a statistical approach for METs to enable selection of parents and best performing lines	Stage 2 & 3 clones at 6 locations across 2 years (2002 to 2003)	FALMM & pedigree	Oakey et al., 2007
Lentils, GY	Y	Explore the AMMI model for selection	MET dataset comprising 11 varieties evaluated at 7 locations over 2–3 years	AMMI	Sabaghnia et al., 2008
Canola, GY & Oil	Y	Develop tools to explore G×E	MET comprising 19 trials in advanced stage of breeding across southern Australian during 2007 to 2008	FALMM & pedigree	Beeck et al., 2010; Cullis et al., 2010
Maize, GY	Y	Investigate G×E on selection decisions	MET comprising, 12 varieties and 25 environments evaluated in 2 consecutive years	AMMI	Perez-Elizalde et al., 2012
Pine Breeding, SD	Y	Present an approach for investigating additive G×E in an outcrossing plant species	77 trials, located across Australia & New Zealand with planting dates spanning the period 1968 to 2005	ARAM & FALMM & pedigree	Cullis et al., 2014
Wheat, 21 traits	Y	Evaluate methods to obtain reliable estimates of variance components	MET comprising single cycle of breeding nurseries: 466 unique locations across 30 years	VCLMM	Arief et al., 2015
Sugarcane, 3 traits	Y	Characterize the varieties in the final selection stage of breeding	MET dataset comprising four consecutive variety series (S00, S03, S04, and S05) planted in the years 2011 to 2014 at 7 locations	VCLMM	Guilly et al., 2017
Pine Breeding, SD	Y	Obtain predicted estimated breeding values for parental selection	107 trials planted across the years 1968 to 2005	FALMM & pedigree	Smith and Cullis, 2018
Lodgepole pine, TH	Y	To model patterns of G×E for a tree breeding program	MET dataset comprising 28 second generation progeny test locations established during 2002 to 2006 across 5 breeding zones	ARAM & FALMM & pedigree	Ukrainetz et al., 2018
Maize, GY	Y	Evaluate the benefit of multi-year and multi-stage data for section	MET comprising stages 1 to 6 across 100 s of locations (some containing multiple trials) across 6 years	2 stage FALMM	Arief et al., 2019

*The Selection column indicates if the aim of the paper was selection on the given dataset. GY, Grain Yield; SD, Stem Diameter; TH, Tree Height; AMMI, Additive Main effect and Multiplicative Interaction; ARAM, Approximate Reduced Animal Model; FALMM, Factor Analytic Linear Mixed Model; LMM, Linear Mixed Model; VCLMM, Variance Component Linear Mixed Model*.

The inherent structure of the breeding program (stages within a year) and thus selection on a stage basis often results in the analysis of plant breeding datasets comprising a series of single year based analyses (Arief et al., 2019). Bernal-Vasquez et al. (2017) finds similarly that breeding program datasets are often analyzed on a year basis and not over years, due to the reasons: that it is simpler/faster and that it is difficult to estimate variation across years due to the lack of common lines between breeding stages. However, due to the commercial nature of plant breeding programs, there is limited literature on the dataset construction of plant breeding METs, within the context of a commercial breeding operation.

One of the central aims of plant breeding programs is to select high yielding and stable genotypes across environments. This is why plant breeding programs evaluate lines over a large number of locations and years as it enables estimates of random occurring cycles of normal and extreme conditions in the TPE (Rosielle and Hamblin, 1981). In the Australian context large and complex G×E has been reported specifically for wheat breeding (Bänziger and Cooper, 2001). For barley, wheat, oats, lupins, peas, lentils, and canola Cullis et al. (2000) found that the main sources of G×E are what they define as “non-static,” that is linked to seasonal influences. This alone contributed to 41% of the total variance. Frensham et al. (1998) similarly found with a Southern Australian oat breeding program that the genotype by year by location (G×Y×L) variance component accounted for 41.1% of the total phenotypic variance. Arief et al. (2015) summarized multiple plant breeding studies, finding that the G×Y×L variance component as a proportion of total phenotypic variance was 29% for wheat and 25% for navy beans breeding programs in Australia. Therefore, there is a need for multiple years of data to accurately quantify genotype performance in the presence of substantial G×E in order to make accurate selection decisions. This is clearly not addressed by single year/single stage based data analysis in a plant breeding program.

The aim of this paper is to demonstrate the utilization of the CG concept for dataset construction. The basic premise is to include sufficient trials to optimize the amount of data on the lines under consideration for selection. By tracing CGs across stages and years it is possible to form a MET dataset with the desired properties. In order to quantify the impact of this approach we use the A-optimality criterion from model-based design theory. The paper is arranged as follows. Section 2 outlines the methodology for MET dataset construction using CGs. The use of A-optimality for comparing datasets is described. In section 3 the methods are applied to two motivating examples from Australian plant breeding programs. Some concluding remarks are given in section 4.

## 2. Methods for MET Dataset Construction

The CG concept for MET data construction is first illustrated using a hypothetical breeding program with four stages of testing (S1 to S4) and in which lines progress through stages without fast-tracking (skipping stages) or retention (remaining in a stage for more years of testing). The aim is to construct a dataset to enable accurate selection decisions for 2018. First consider the decisions on lines in S4 in 2018. These lines commenced their testing in S1 trials in 2015 (so are all members of CG15), were selected to be tested in S2 trials in 2016, then S3 trials in 2017, and finally S4 trials in 2018. Thus, in order to capture all of the data on the lines under consideration for selection, we would combine data from all of the trials in this sequence. Overall, this would suggest a separate analysis for each of the selection decisions, based on combining data from the following trials:

- Selection decision S1: S1 trials 2018
- Selection decision S2: S2 trials 2018 + S1 trials 2017
- Selection decision S3: S3 trials 2018 + S2 trials 2017 + S1 trials 2016
- Selection decision S4: S4 trials 2018 + S3 trials 2017 + S2 trials 2016 + S1 trials 2015.

It is instructive to illustrate this compilation of trials across stages and years using tables such as Table 2. In this table the diagonal bands of stages across years are labeled as A to I, with the labels A to D being assigned in such a way that they align with the S1 to S4 trials in the year of selection (here 2018). The datasets described above correspond to the diagonal bands of trials labeled as A to D. Thus, for example, band D comprises data from S1 trials in 2015, S2 trials in 2016, S3 trials in 2017, and S4 trials in 2018. It is important to note that, for any given trial, the data from all of the harvested plots is included and not just the data on the lines of interest. Thus, for example, the data from S1 trials in 2015 relates to all of the lines tested in those trials. We describe bands A to F in Table 2 as being “complete” in the sense that they trace back to the first stage of testing, namely S1. In contrast, bands G to I are incomplete, with band G missing S1 trials, band H missing S1 and S2 trials and band I missing S1, S2, and S3 trials. This has implications in terms of selection bias which will be discussed in section 3.

**Table 2:** Data bands for potential inclusion in a MET dataset for selection decisions in 2018 from a breeding program with four stages of selection (S1 to S4).

	**Year**					
**Stage**	**2013**	**2014**	**2015**	**2016**	**2017**	**2018**
S1	F	E	D	C	B	A
S2	G	F	E	D	C	B
S3	H	G	F	E	D	C
S4	I	H	G	F	E	D

*Data bands correspond to diagonal sequences of stages across years and are labeled as A to I*.

In the absence of retention or fast-tracking, all the lines in S1, S2, S3, and S4 in 2018 are members of CG18, CG17, CG16, and CG15, respectively. Thus, all the lines within a stage in 2018 belong to a single CG only and the entire selection history for any of these lines is captured in the associated data band. The generalization to more complex scenario will be discussed in the context of the motivating examples (see section 3).

In terms of information available for each of the four selection decisions it is instructive to differentiate between “direct” and “indirect” information. The former relates to observed data so is maximized by including all trials in which the lines of interest have been grown. In the hypothetical example this corresponds to the bands so suggests the conduct of four analyses each based on a separate band (A, B, C, and D). However, the use of a FALMM for analysis creates the possibility of also using indirect information derived from genetically related lines in other trials. We would therefore recommend undertaking a single analysis using data combined across these bands. This recommendation can be justified by applying the method described in section 2.1 to quantify information for selection. Finally, we note that in the MET analysis, G×E is modeled with reference to environments which are defined to be combinations of trial locations and years. Combining across bands may lead to the presence of multiple trials at a single location within a year. For example, in any given year, locations with S1 trials also typically include S2, S3, and S4 trials. We refer to such trials as “co-located.” This will be discussed further in section 3.

### 2.1. Quantifying Information for Selection in MET Datasets

In order to discriminate among possible MET datasets in terms of the amount of information available for selection decisions, we note that the problem has strong links with optimal (model-based) design. As Butler et al. (2014) state, “The goal of optimal design is to discriminate among competing designs in an effort to maximize the treatment information from a fixed number of experimental units.” This requires the use of an optimality criteria, and, in the context of plant breeding trials in which the treatments are genotypes and the aim is selection, the A-optimality criteria is appropriate since this aligns with minimizing the probability of an incorrect selection decision (Bueno Filho and Gilmour, 2003). A-optimality is based on the so-called A-value which is the average pairwise variance of elementary treatment contrasts. We therefore propose to use A-values to quantify the treatment (genotype) information available in any given MET dataset.

In model-based design, A-values are computed under a pre-specified Linear Mixed Model (LMM) which we will term the design model. Specification of the design model requires specification of the fixed and random effects, the variance models for the random effects and residuals and the values of the associated variance parameters. The design model is usually chosen to be as close as possible to that expected for the analysis. Additionally, the variance parameter values are chosen as being “typical” so may be based on historic analyses. The model proposed in this paper for the analysis of MET data is the FALMM with the inclusion of pedigree information. Genotype selections using this model are typically focused on the measure of overall genotype performance (across environments) as presented in Smith and Cullis (2018). However, the factor analytic variance parameters are specific to the individual environments in the dataset so that typical values do not exist. Therefore a more generic, but still realistic design model is required for assessing MET dataset information. We have chosen a variance component model that involves random genotype main effects and random G×E effects, both of which are partitioned into additive and non-additive effects. This is, in fact, a sub-model of the FALMM. The A-values are then computed for the total (additive plus non-additive) genotype main effects since these provide a measure of average performance of genotypes across environments.

In order to determine reasonable values for the variance parameters in this design model we consider Cullis et al. (2000) who conducted variance component analyses of grain yield in 22 MET datasets from Australian crop variety evaluation programs. The environments in those datasets were classified according to the year, the geographic region and possibly location within region so Cullis et al. (2000) partitioned G×E accordingly. In our motivating examples we do not have regional information nor are trials typically located in identical positions from year to year. However, we recognize the importance of genotype by year interaction so maintain this as a separate source in the design model. Thus we have used the genotype main effects (G), genotype by year interaction (G×Y), and Error sources of variation from Cullis et al. (2000), and have added together the remaining sources to form residual genotype by environment interaction. The mean percentage contributions for each of these sources across all 22 datasets was 13.77% (G), 8.59% (G×Y), 37.91% (residual G×E), and 39.73% (Error) (see Table 3). In the model-based design literature, and without loss of generality, a value of one is typically assumed for the error variance. We adopt the same approach here (see second row in Table 3).

**Table 3:** Variance parameter values for design model for genotype main effects (G), genotype by year interaction (G×Y), residual genotype by environment interaction (G×E), and Error.

	**G**	**G×Y**	**G×E**	**Error**
Mean % from Cullis et al. (2000)	13.77	8.59	37.91	39.73
Total variance parameter	0.35	0.22	0.95	1.00
Additive variance parameter	0.28/ā	0.18/ā	0.76/ā	
Non-additive variance parameter	0.07	0.04	0.19	

*Rows in the table are: means of percentages in Cullis et al. (2000); associated (total) variance parameter values assuming error variance of one; additive variance parameter values (numerator is 80% of total values and denominator, ā, is the mean of the diagonal elements of NRM); non-additive variance parameter values (20% of total values)*.

In contrast to our approach, the analyses in Cullis et al. (2000) do not involve information on genetic relatedness. We therefore make the further assumption that additive variance comprises 80% of total variance. This represents an average from the analyses of numerous Australian plant breeding datasets. The final values for the variance parameters in the design model are given in the third and fourth rows of Table 3. All A-values in this paper were computed using ASReml-R (Butler et al., 2017). The code is provided in Appendix A.

## 3. Results

In this section we show the application of the methods presented in section 2 to two motivating examples.

### 3.1. Durum Breeding Program

Durum wheat (*Triticum durum desf*.) breeding in Australia is currently resourced on agronomic zones of production and funded by New South Wales Department of Primary Industries (NSW DPI), The University of Adelaide and the Grains Research and Development Corporation (GRDC) under the Durum Breeding Australia (DBA) project. The motivating example of our paper is the DBA North program that operates out of Tamworth Agricultural Institute, capturing TPEs in New South Wales (NSW) up to and including central Queensland (QLD). The structure of the program is illustrated in Figure 1. The S1 in any year contains on average 1120 lines evaluated in one or two environments. As the program progresses the line numbers decrease to on average, 60 lines in the S4, evaluated in eight environments.

The aim of this paper is to construct a dataset to evaluate the performance of the 2018 S1, S2, S3, and S4 lines for selection and progression to the next stage of testing. The data available for this purpose spanned the period 2013 to 2018 so the sequences of stages and years (and thence data bands) are the same as shown in Table 2. The actual numbers of trials in each stage and year, and the total numbers of trials in each complete data band, are given in Table 4. Note that there were co-located trials within stages in many environments so that the numbers of environments is also provided in this table. The numbers of test lines for each stage and year (2013 to 2018) are given in Table 5. Note that test lines refer only to the lines under consideration for selection, so excludes check varieties, for example. At any stage of selection, a line may be selected to progress to the next stage of testing, retained in the same stage or rejected. In contrast to the hypothetical example, lines are often retained within later stages for additional year/s of testing. Retentions may occur due to limitations in seed production, or even a holding pattern while awaiting disease and/or quality data. This has resulted in the lines in later stages comprising a mixture of CGs. The distribution across CGs for 2018 lines are given as the final columns in Table 5. For example, the majority (66) of lines in S3 in 2018 correspond to CG16 so have followed the simple progression along band C (that is, they progressed from S1 trials in 2016 to S2 trials in 2017 to S3 trials in 2018). But a fair number (22) correspond to CG15 and progressed from S1 trials in 2015 to S2 trials in 2016 to S3 trials in 2017 and were then retained in S3 in 2018. Finally, five lines correspond to CG14 and progressed from S1 trials in 2014 to S2 trials in 2015 to S3 trials in 2016 and were then retained in S3 in 2017 and 2018. This has implications for construction of the MET dataset.

**Table 4:** Number of trials and environments (presented as trials/environments) in each stage (S1 to S4) and year (2013-2018) in the durum breeding program with data bands indicated as superscripts.

	**Year**						**Band**
**Stage**	**2013**	**2014**	**2015**	**2016**	**2017**	**2018**	**Totals**
S1	2/1^*F*^	5/2^*E*^	6/1^*D*^	6/1^*C*^	7/1^*B*^	6/1^*A*^	6/1^*A*^
S2	6/3^*G*^	8/2^*F*^	8/2^*E*^	3/1^*D*^	3/1^*C*^	6/2^*B*^	13/3^*B*^
S3	4/4^*H*^	3/3^*G*^	5/4^*F*^	3/3^*E*^	3/3^*D*^	3/3^*C*^	12/5^*C*^
S4	9/9^*I*^	8/8^*H*^	12/10^*G*^	6/5^*F*^	11/10^*E*^	6/6^*D*^	18/11^*D*^
							27/17^*E*^
							21/12^*F*^

*The final column gives the total numbers of trials and environments in each of the complete data bands (A to F)*.

**Table 5:** Number of test lines in each stage (S1 to S4) and year (2013-2018) in the durum breeding program.

	**Number of test lines**						**Number of 2018 test lines**					
**Stage**	**2013**	**2014**	**2015**	**2016**	**2017**	**2018**	**CG18**	**CG17**	**CG16**	**CG15**	**CG14**	**CG13**
S1	582	1485	1000	1163	1303	1148	1148	0	0	0	0	0
S2	105	361	413	388	379	315	0	315	0	0	0	0
S3	30	92	92	92	90	93	0	0	66	22	5	0
S4	25	41	57	55	53	56	0	0	0	31	12	13

*The final columns give the number of lines in each contemporary group (CG18-CG13) for lines under consideration for selection in 2018*.

The starting point for MET dataset construction for selection decisions on the 2018 lines (S1 to S4) involves all trials in bands A-D as described in the hypothetical example. With the retention of lines it is clear that this would fail to capture much of the data on the 30 S3 and S4 lines in 2018 that belonged to CG14 and CG13 (see Table 5). For example, Table 6 shows there are nine lines in S4 for which 5 years of data would be missing if the dataset comprised only bands A-D; another nine lines for which 4 years would be missing and a further seven lines missing 2 or 3 years of data. We believe this is unacceptable. We therefore investigate the addition of bands E and F to the data. Table 6 shows the improvement in capturing more of the data on the lines of interest. Full data on the lines of interest could be obtained by adding band G but we caution against this because band G is incomplete (it is missing S1 trials, see Table 2) so we do not have the entire selection history for many of the lines in band G. The inclusion of band G, or indeed the other incomplete bands H and I (so that the entire rectangle of data is included) may result in “selection bias,” that is, bias in the estimates of the genetic variance parameters (Thompson, 1973) so is not recommended. The final dataset is therefore chosen to comprise bands A-F. With this dataset only five lines under scrutiny for selection (in S4) are missing data and the amount missing is small (1 or 2 years out of a total of 6 years).

**Table 6:** MET dataset construction for 2018 selection decisions in durum breeding program.

	**# years**	**Bands in dataset**			
**Stage**	**missing**	**A-D**	**A-E**	**A-F**	**A-G**
S1	0	1148	1148	1148	1148
S2	0	315	315	315	315
S3	0	88	93	93	93
	2	5	0	0	0
S4	0	31	42	51	56
	1	0	1	3	0
	2	6	0	2	0
	3	1	0	0	0
	4	9	13	0	0
	5	9	0	0	0

*Number of lines missing years of data in datasets comprising bands A-D, A-E, A-F, and A-G. Total number of lines for selection: 1148, 315, 93, and 56 (for stages S1 to S4)*.

The final MET dataset (bands A-F) for analysis comprised yield data on 6,951 lines from 21,660 plots corresponding to 97 trials (see Table 4) across 30 environments. Each field trial was sown as a rectangular array indexed by field rows and columns. Trials were sown in a serpentine sequence and harvested in the row direction with all other management regimes applied via pathways in the column dimension. Trials were designed as grid-plot, partially replicated (*p*-rep) (Cullis et al., 2006) or randomized complete block designs, with two to three replicates. Summary information for the 30 environments is given in Table 7. There were 15 environments with co-located trials, ranging in number from two to 13. The co-located trials related either to different stages or to multiple trials within stages (also see Table 4), the latter being due to physical restrictions on trial sizes. We note that co-located trials are only deemed to comprise a single environment when they are all managed in the same way, that is, they are sown and harvested within a similar time frame and subjected to the same agronomy practices including fertilizer, herbicide, and pathway regimes.

**Table 7:** Summary of environments in the durum MET dataset: number of trials for each stage of testing (S1, S2, S3, S4) and total number of trials.

	**Number of trials**							**Mean**
**Environment**	**S1**	**S2**	**S3**	**S4**	**Total**	**nplot**	**nline**	**yield**
2013-Breeza	2	0	0	0	2	720	585	2.88
2014-Breeza	3	0	0	0	3	1296	937	4.28
2014-Edgeroi	0	4	0	0	4	768	364	2.78
2014-Tworth	2	4	0	0	6	1468	915	3.93
2015-Breeza	0	0	2	0	2	384	96	5.15
2015-Edgeroi	0	4	1	0	5	1056	498	1.83
2015-Nstar	0	0	1	0	1	192	96	5.05
2015-Tworth	6	4	1	0	11	2244	1499	4.00
2016-Breeza	0	0	1	1	2	372	152	4.35
2016-Edgeroi	0	0	0	1	1	180	60	4.79
2016-Gurley	0	0	0	1	1	180	60	5.62
2016-Nstar	0	0	1	2	3	552	152	5.49
2016-Tworth	6	3	1	1	11	2628	1704	4.81
2017-Blbgra	0	0	0	1	1	180	60	1.12
2017-Breeza	0	0	1	1	2	384	158	5.31
2017-Bribbaree	0	0	0	1	1	180	60	1.20
2017-Coonamble	0	0	0	1	1	180	60	1.61
2017-Edgeroi	0	0	0	1	1	180	60	3.93
2017-Garah	0	0	0	1	1	180	60	1.84
2017-Gurley	0	0	0	1	1	180	60	2.12
2017-Nstar	0	0	1	1	2	384	158	3.41
2017-Tworth	7	3	1	2	13	3014	1836	4.26
2017-Westmar	0	0	0	1	1	180	60	2.24
2018-Blbgra	0	0	0	1	1	198	66	1.24
2018-Breeza	6	3	1	1	11	2502	1629	5.53
2018-Coonamble	0	0	0	1	1	198	66	1.55
2018-Gurley	0	0	1	0	1	210	105	2.23
2018-Moree	0	0	0	1	1	198	66	1.51
2018-Trangie	0	0	0	1	1	198	66	1.02
2018-Tworth	0	3	1	1	5	1074	481	2.24

*Number of plots (nplot) and lines (nline); mean yield (t/ha)*.

The pedigree information associated with the above trial data contained 7,628 records. All lines in the MET data set had pedigree information. This was the first time a pedigree file had been created for this breeding program and included in the analysis. This was a significant undertaking as the durum breeding program was established in the 1960's and a comprehensive pedigree file (outside annual crossing block information) had not existed in the program since this time. The NRM was formed using the *pedicure* package (Butler, 2019) in R (R Development Core Team, 2015). The inbreeding coefficients of lines with phenotypic data ranged from 0.750 to 0.998 with mean of 0.905.

**Figure 2:**
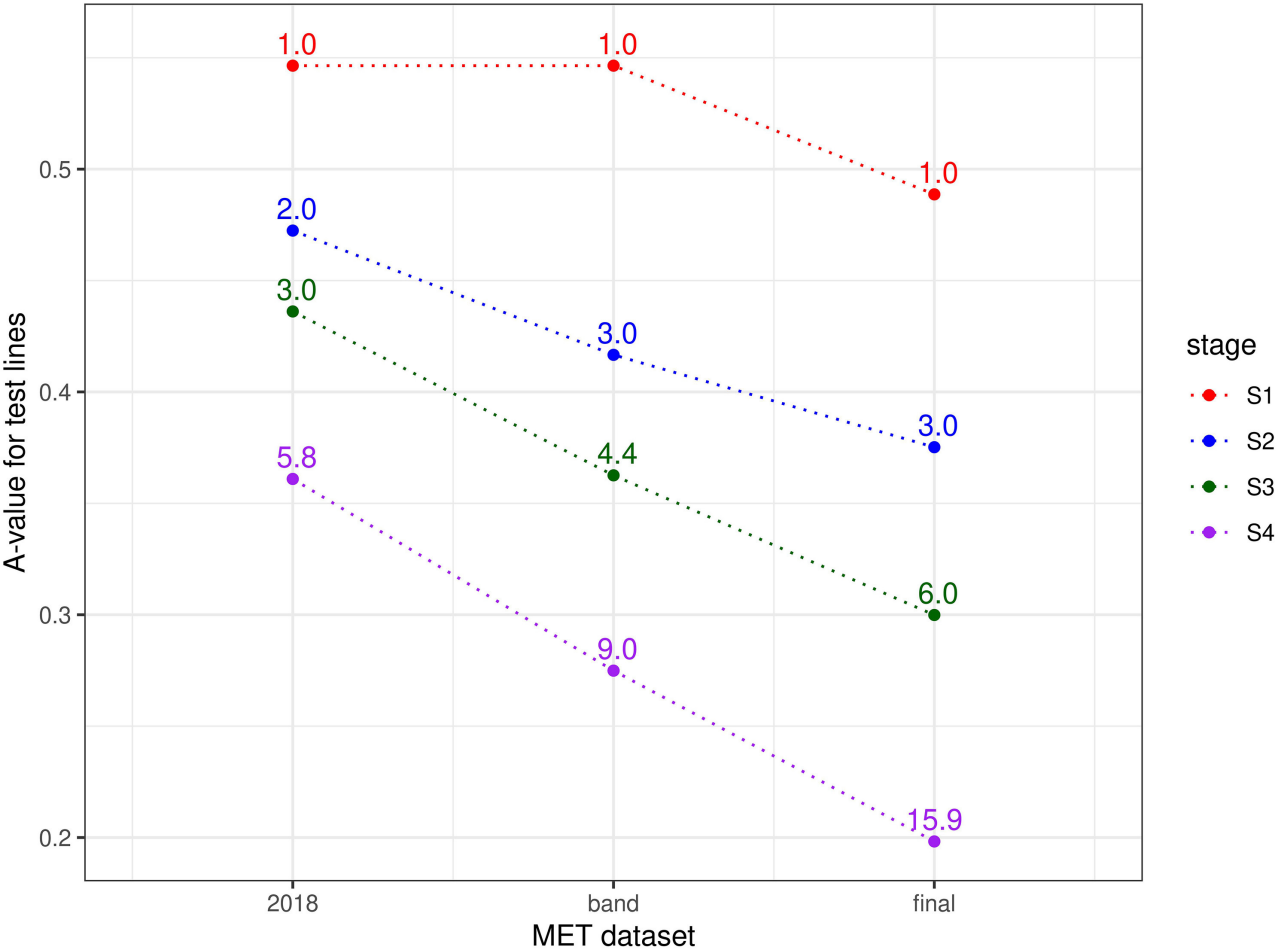
A-values for lines under consideration for selection in durum breeding program in 2018 (stage S4: 56 lines, S3: 93 lines, S2: 315 lines, and S1: 1148 lines). A-values are given for three types of MET dataset, namely 2018 data for each stage; diagonal band of data for each stage and the final dataset constructed as in section 3.1. The points are labeled with the associated mean numbers of environments in which these lines were grown. Note that for S1 the 2018 and band datasets are the same.

Finally, the approach described in section 2.1 for comparing MET datasets in terms of the information for selection was applied for each stage of selection and for three types of MET dataset, namely the 2018 data for each stage, the diagonal band of data for each stage and the final dataset (bands A-F). Thus, for S4 selections, the three datasets comprised data from S4 trials in 2018 alone; data from trials in band D (S4 trials in 2018 + S3 trials in 2017 + S2 trials in 2016 + S1 trials in 2015) and the final dataset. For S3 selections, the three datasets comprised data from S3 trials in 2018 alone; data from trials in band C (S3 trials in 2018 + S2 trials in 2017 + S1 trials in 2016) and the final dataset. For S2 selections, the three datasets comprised data from S2 trials in 2018 alone; data from trials in band B (S2 trials in 2018 + S1 trials in 2017) and the final dataset. Note that for S1 selections, the single year dataset (S1 trials in 2018) is equivalent to the band dataset (band A). The resultant A-values are shown in Figure 2. This clearly shows the superiority of the final dataset in each case. The reduction in A-values is largely driven by an increase in the amount of direct information (as reflected in the mean numbers of environments per line) but there is also a strong contribution from indirect information. For example, the S1 lines under consideration for selection in 2018 were only grown in a single environment (see Table 4) so there is no difference in direct information between using the 2018 data alone for this stage compared with the final dataset. However, the A-value for the final dataset is much lower, indicating the impact of indirect information from relatives of the S1 lines.

### 3.2. Chickpea Breeding Program

In Australia, the chickpea (*Cicer arietinum* L.) breeding program is managed under the umbrella of Pulse Breeding Australia (PBA). PBA is an Australian government funded project through the Grains Research and Development Corporation (GRDC). PBA coordinates and funds the breeding activities of the four pulse crops of economic importance—chickpeas, field peas, faba beans, and lentils across the Australian growing environment. The chickpea program under PBA comprises three sub-programs, which are based on the following germplasm streams—desi (north and south) and kabuli. We will focus on the desi south sub program managed by NSW DPI. This program evaluates lines annually, across southern NSW, Victoria and South Australia in three stages, namely S1, S2, and S3 across multiple locations. The structure of the program is similar to that of the program illustrated in Figure 1. The S1 in any year contains on average 650 lines evaluated across five environments. As the program progresses the line numbers decrease to on average, 70 lines in S3, evaluated in eight environments.

Here we consider the MET dataset construction to evaluate the performance of test lines in all three stages in 2019. The data available for this purpose spanned the period 2016 to 2019. The sequences of stages and years (and thence data bands), together with their associated numbers of trials are given in Table 8. Note that there were no co-located trials within stages so that the numbers of environments in the cells of this table are the same as the numbers of trials. The numbers of test lines for each stage and year (2016 to 2019) are given in Table 9. The distribution across CGs for 2019 lines are given as the final columns in Table 9. Thus, for example, the majority (46) of lines in S3 correspond to CG17 (so have followed the simple progression along band C) but 11 correspond to CG16 and one to CG14.

**Table 8:** Number of trials in each stage (S1 to S3) and year (2016-2019) in the chickpea breeding program with data bands indicated as superscripts.

	**Year**				**Band**
**Stage**	**2016**	**2017**	**2018**	**2019**	**Totals**
S1	4^*D*^	5^*C*^	5^*B*^	6^*A*^	6^*A*^
S2	4^*E*^	6^*D*^	6^*C*^	7^*B*^	12^*B*^
S3	5^*F*^	8^*E*^	8^*D*^	9^*C*^	20^*C*^
					18^*D*^

*The final column gives the total numbers of trials in each of the complete data bands (A to D)*.

**Table 9:** Number of test lines in each stage (S1 to S3) and year (2016-2019) in the chickpea breeding program.

	**Number of test lines**				**Number of 2019 test lines**					
**Stage**	**2016**	**2017**	**2018**	**2019**	**CG19**	**CG18**	**CG17**	**CG16**	**CG15**	**CG14**
S1	443	559	763	638	633	5	0	0	0	0
S2	113	100	146	176	0	176	0	0	0	0
S3	58	58	49	58	0	0	46	11	0	1

*The final columns give the number of lines in each contemporary group (CG19-CG14) for lines under consideration for selection in 2019*.

The starting point for MET dataset construction for selection decisions on the 2019 lines (S1 to S3) involves all trials in bands A-C. However, this would fail to capture all the data on the 11 S3 lines in 2019 that belonged to CG16 and the single line that belonged to CG14 (also see Table 10). Addition of band D accommodates the former and provides an additional year for the latter. Full data on the lines of interest could be obtained by adding bands E and F but once again we caution against this because these bands are incomplete so would introduces many lines with incomplete selection histories. The final dataset is therefore chosen to comprise bands A-D. With this dataset only a single line (in S3) is missing data. We note that this line has been retained in S3 for a number of years and is of less interest in terms of promotion to the next stage of testing (in the NVT).

**Table 10:** MET dataset construction for 2019 selection decisions in chickpea breeding program.

	**# years**	**Bands in dataset**			
**Stage**	**missing**	**A-C**	**A-D**	**A-E**	**A-F**
S1	0	638	638	638	638
S2	0	176	176	176	176
S3	0	46	57	57	58
	1	11	0	1	0
	2	0	1	0	0
	3	1	0	0	0

*Number of lines missing years of data in datasets comprising bands A-C, A-D, A-E, and A-F. Total number of lines for selection: 638, 176, and 58 (for S1 to S3)*.

The final MET dataset (bands A-D) for analysis comprised yield data on 2,448 lines from 18,936 plots corresponding to 56 trials (see Table 8) across 28 environments. Summary information for the 28 environments is given in Table 11. There were 18 environments with co-located trials, with between two to three trials. These were all associated with different stages within environments.

**Table 11:** Summary of environments in the chickpea MET dataset: number of trials for each stage of testing (S1, S2, S3) and total number of trials.

	**Number of trials**						**Mean**
**Environment**	**S1**	**S2**	**S3**	**Total**	**nplot**	**nline**	**yield**
2016-Balaklava	1	0	0	1	504	440	1.78
2016-Horsham	1	0	0	1	504	436	2.17
2016-Melton	1	0	0	1	504	450	2.17
2016-Yenda	1	0	0	1	504	360	2.59
2017-Balaklava	1	1	0	2	744	510	0.97
2017-Curyo	0	1	0	1	216	108	1.78
2017-Horsham	1	1	0	2	756	508	2.22
2017-Melton	1	1	0	2	756	512	1.36
2017-Yenda	1	1	0	2	924	648	1.18
2017-York	1	1	0	2	480	312	1.71
2018-Ardlethan	1	1	1	3	1356	972	0.66
2018-Balaklava	1	1	1	3	1032	676	0.44
2018-Curyo	0	1	1	2	492	210	0.74
2018-Horsham	1	1	1	3	948	599	2.02
2018-Melton	1	1	1	3	1032	676	0.49
2018-Mingenew	1	1	1	3	876	522	1.42
2018-Northampton	0	0	1	1	192	65	1.62
2018-Wagga Wagga	0	0	1	1	192	65	1.31
2019-Ardlethan	1	1	1	3	1260	858	0.35
2019-Curyo	0	1	1	2	444	180	1.63
2019-Dalwallinu	0	1	1	2	456	182	0.23
2019-Horsham	1	1	1	3	984	594	1.26
2019-Melton	1	1	1	3	816	414	0.59
2019-Mingenew	1	1	1	3	948	570	0.99
2019-Narrabri	1	0	0	1	780	642	2.11
2019-Pinery	1	1	1	3	756	368	0.78
2019-Wagga Wagga	0	0	1	1	240	81	0.58
2019-Yenda	0	0	1	1	240	80	1.38

*Number of plots (nplot) and lines (nline); mean yield (t/ha)*.

The pedigree information associated with the above trial data contained 2,983 records. All lines in the MET data set had pedigree information. The inbreeding coefficients of lines with phenotypic data ranged from 0.500 to 0.999 with mean of 0.7404.

**Figure 3:**
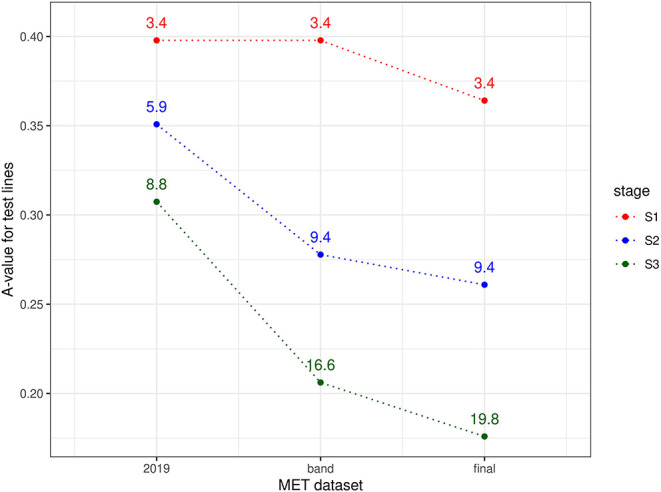
A-values for lines under consideration for selection in chickpea breeding program in 2019 (stage S3: 58 lines, S2: 176 lines, and S1: 638 lines). A-values are given for three types of MET dataset, namely 2019 data for each stage; diagonal band of data for each stage and the final dataset constructed as in section 3.2. The points are labeled with the associated mean numbers of environments in which these lines were grown. Note that for S1 the 2019 and band datasets are the same.

The information available for selection at each stage was assessed using a similar approach to the durum example, namely computing A-values for test lines for three types of MET dataset. For S3 selections, the three datasets comprised data from S3 trials in 2019 alone; data from trials in band C (S3 trials in 2019 + S2 trials in 2018 + S1 trials in 2017) and the final dataset (trials in bands A-D). For S2 selections, the three datasets comprised data from S2 trials in 2019 alone; data from trials in band B (S2 trials in 2019 + S1 trials in 2018) and the final dataset. Note that for S1 selections, the single year dataset (S1 trials in 2019) is equivalent to the band dataset (band A). The resultant A-values are shown in Figure 3. Once again the superiority of the final dataset in each case is clearly shown, with greatly reduced A-values compared to the single year and single band datasets.

## 4. Discussion

In this paper we have addressed a void in the literature, and provided a rigorous framework for the construction of MET datasets for selection in plant breeding programs. We have described a method that aims to optimize the amount of information available on the “lines of interest,” that is, the lines under consideration for selection. The method is intuitive and involves several simple steps. A key aspect is to identify contemporary groups (CGs), that is, groups of lines that entered the first stage of testing (S1) in the same year. This allows a complete enumeration of the trials in which the lines of interest have been grown. In addition to defining CGs, which relate to lines, we have also introduced the concept of data bands, which relate to trials. Data bands align with the testing system, namely the progression through stages (for example S1 through to S4) from year to year. By sequentially building the MET dataset using data bands we can form a dataset that captures as much information as possible for the lines of interest.

We have also developed a method for quantifying this information for any given MET dataset. The method uses fundamental concepts from model-based design theory. Thus, information is quantified using an A-value (average pairwise variance) for the lines of interest from a pre-specified linear mixed model. The application of this approach to the two motivating examples in this paper clearly showed the superiority of the MET datasets constructed using the CG approach, in particular when compared with the more common approach of simply using trials from a single stage and year. The information gains for all selection stages were associated both with direct information, that is, from the trials in which the lines of interest were grown and also indirect information derived from trials in which genetically related lines were grown.

MET datasets constructed using the CG approach encompass multiple bands of data and this suggests a further and crucial gain for S1 selection decisions since these lines have only been grown in a single year and often at only one or two locations. The key driver here is that locations with S1 trials also typically include later stage trials. The environment is then defined as the amalgamation of all of these (co-located) trials and thence includes a far wider set of lines than the S1 lines alone. This provides links between environments (both within and between years) for the S1 lines. Then application of the FALMM will allow examination of G×E for the S1 lines of interest across a wide range of environments. This is particularly important in an Australian context as genotype by location by year interaction is often the largest component of G×E (Frensham et al., 1998; Cullis et al., 2000). Hence the additional seasons of data will lessen the impact of selection on a single year of data, which could be a seasonally extreme year and therefore outside what is expected for the range of TPE. This issue has also been pointed out by Arief et al. (2015).

We note that combining trials utilizing the CG approach may result in an unbalanced dataset, with poor connectivity (low numbers of lines in common) between some environments. Whilst one of the advantages of using an FALMM is the ability to handle unbalanced data, there has been concern about the reliability of estimation of FA parameters in extreme cases when connectivity is very poor. It is well-known that poorly estimated genetic variance parameters will result in a reduction in genetic gain (Sales and Hill, 1976a,b). The A-value approach does not take this into account since the variance parameters are assumed known. Historically, genotype connectivity was thought to be the key determinant of the reliability of estimation of FA parameters. We are currently developing a formal information based diagnostic for this purpose. It is superior to connectivity in the sense of better forecasting the uncertainty of variance parameter estimates and being applicable for both additive and non-additive genetic variance parameters. It may therefore be applied jointly with the A-value approach in order to balance genotype information and reliability of variance parameter estimation in the search for an optimum MET dataset.

The applications in this paper are based on inbred annual crops. However, we note that our methods can be easily modified for situations that include hybrid crops, which require evaluation of inbred parental lines in addition to the hybrids themselves. The methods are also applicable for parental evaluation in perennial crops such as radiata pine, in which parental trees are evaluated by the performance of their progeny in (field) trials across numerous years and seasons (Smith and Cullis, 2018). We note that all models considered in this paper partitioned genetic effects into additive and non-additive effects. This was achieved with the use of a numerator relationship matrix formed from pedigree (ancestral) records. It would be straight-forward to replace this with a genomic relationship matrix formed from marker data.

Finally, and at the request of the editor, we note that an important area of research that requires MET datasets is the investigation of genotype by environment by management (G×E×M) interactions. Our methodology was developed in the context of (standard) two-way G×E studies but could be generalized to three-way G×E×M studies using, for example, linear mixed models of the form presented in Smith et al. (2019). In Smith et al. (2019) the management practice was the application of a fungicide to canola seed in order to limit the impact of the disease blackleg. Within environments, the treatments comprised the factorial combinations of genotypes and the presence/absence of the fungicide and the experimental designs included replication for both factors. This allowed valid inference on G×E×M interactions. Unfortunately this is rarely the case for G×E×M studies, with the management treatment often not having proper replication. A common example is studies involving the presence/absence of an abiotic stress such as heat or drought. It is often argued that the nature of the equipment required for application of the stress factor is such that replication is not possible. This means that inference on G×E×M interactions is compromised. Kadkol et al. (2020) discuss this in detail. We argue that it should be possible to construct experimental designs that allow a limited, but sufficient amount of replication for stress treatments so that valid inference on G×E×M interactions is possible. Given the cost and importance of such research, this would seem a priority.

Our methodology for constructing a MET dataset has been programmed within the R computing environment (R Development Core Team, 2015) and the code is available upon request. The method for quantifying information involves the use of ASReml-R (Butler et al., 2017) and example code is given in Appendix A. We have implemented these methods in a number of plant breeding programs with the result of superior MET datasets and also improved breeder confidence and understanding.

## Data Availability Statement

The datasets presented in this article are not readily available because they are owned by New South Wales Department of Primary Industries and the Grains Research and Development Corporation. Requests to access the datasets should be directed to gururaj.kadkol@dpi.nsw.gov.au (durum data) and kristy.hobson@dpi.nsw.gov.au (chickpea data).

## Author Contributions

BC and AS conceived the ideas and developed the methodology. AS wrote all sections of the manuscript apart from the Introduction which was written by AG. AG and CL prepared and curated the datasets. GK and KH provided the data and input on plant breeding perspectives. AS and CL performed the statistical calculations. BC and CL contributed to manuscript revision. All authors read and approved the submitted version.

## Conflict of Interest

AG commenced employment with InterGrain when this manuscript was nearing completion. The remaining authors declare that the research was conducted in the absence of any commercial or financial relationships that could be construed as a potential conflict of interest.

## Appendices

### A. Code for Computing A-values

We consider S4 selection decisions for the final MET dataset for the durum motivating example. The data-frame is “final.df” and the key fields in this data-frame are “Environment” (factor with 30 levels); Gkeep (factor with 6,951 levels corresponding to the lines grown in the trials) and “Year” (factor with 6 levels). The inverse of the NRM is stored in sparse form in the object “durum.giv” and corresponds to 7,628 lines (lines grown in the trials and their ancestors). The mean of the diagonal elements of the NRM is ā = 1.9. The steps in ASReml-R (Butler et al., 2017) are as follows:

1. run the model with start.values=T to be able to set the values of the variance parameters
    sv  <- asreml(Yield~Environment, random =~vm(Gkeep, durum.giv) +
    idv(Year) : vm(Gkeep, durum.giv) + idv(Environment) : vm(Gkeep, durum.giv) +
    ide(Gkeep) + Year : ide(Gkeep) + Environment : ide(Gkeep),
    residual=~idv(units), data=final.df, start.values=TRUE,
    na.action = na.method(x='include'))
2. set the variance parameter values as given in Table 3. These are ordered as additive G, G×Y and G×E followed by non-additive G, G×Y and G×E, then Error. Also set constraints so the parameters are fixed at these values in the next run of the model
    gam.all  <- sv$vparameters.table
    gam.all$Value  <- c(0.15, 0.09, 0.40, 0.07, 0.04, 0.19, 1, 1)
    gam.all$Constraint  <- 'F'
3. run the model with the (fixed) pre-specified variance parameter values
    temp.asr  <- asreml(Yield Environment, random =~vm(Gkeep, durum.giv) +
    idv(Year) : vm(Gkeep, durum.giv) + idv(Environment) : vm(Gkeep, durum.giv) +
    ide(Gkeep) + Year : ide(Gkeep) + Environment : ide(Gkeep),
    residual=~idv(units), data=final.df, R.param=gam.all, G.param=gam.all,
    na.action = na.method(x='include'), maxit=1)
4. use the ASReml-R predict function to predict the total (additive + non-additive) line effects for the test lines in S4 in 2018 (there are 56 of these with names stored in the vector gs4.2018). Then obtain the A-value as the average pairwise variance (square of average sed)
    temp.pvs  <- predict(temp.asr, classify='Gkeep', maxit=1,
    only=c("vm(Gkeep, durum.giv)", "ide(Gkeep)"), levels=gs4.2018)
    Avalue  <- temp.pvs$avsed^2
